# Histatin-1 Attenuates LPS-Induced Inflammatory Signaling in RAW264.7 Macrophages

**DOI:** 10.3390/ijms22157856

**Published:** 2021-07-23

**Authors:** Sang Min Lee, Kyung-No Son, Dhara Shah, Marwan Ali, Arun Balasubramaniam, Deepak Shukla, Vinay Kumar Aakalu

**Affiliations:** 1 Department of Ophthalmology and Visual Sciences, University of Illinois at Chicago, Chicago, IL 60612, USA; dna1710@uic.edu (S.M.L.); kson@uic.edu (K.-N.S.); Dshah40@uic.edu (D.S.); marwan@uic.edu (M.A.); arun@uic.edu (A.B.); dshukla@uic.edu (D.S.); 2 Department of Microbiology and Immunology, University of Illinois at Chicago, Chicago, IL 60612, USA; 3 Research and Surgical Services, Jesse Brown VA Medical Center, Chicago, IL 60612, USA

**Keywords:** histatin, lipopolysaccharide, anti-microbial peptide, macrophage, innate, adaptive, immune, inflammation

## Abstract

Macrophages play a critical role in the inflammatory response to environmental triggers, such as lipopolysaccharide (LPS). Inflammatory signaling through macrophages and the innate immune system are increasingly recognized as important contributors to multiple acute and chronic disease processes. Nitric oxide (NO) is a free radical that plays an important role in immune and inflammatory responses as an important intercellular messenger. In addition, NO has an important role in inflammatory responses in mucosal environments such as the ocular surface. Histatin peptides are well-established antimicrobial and wound healing agents. These peptides are important in multiple biological systems, playing roles in responses to the environment and immunomodulation. Given the importance of macrophages in responses to environmental triggers and pathogens, we investigated the effect of histatin-1 (Hst1) on LPS-induced inflammatory responses and the underlying molecular mechanisms in RAW264.7 (RAW) macrophages. LPS-induced inflammatory signaling, NO production and cytokine production in macrophages were tested in response to treatment with Hst1. Hst1 application significantly reduced LPS-induced NO production, inflammatory cytokine production, and inflammatory signaling through the JNK and NF-kB pathways in RAW cells. These results demonstrate that Hst1 can inhibit LPS-induced inflammatory mediator production and MAPK signaling pathways in macrophages.

## 1. Introduction

Responses to environmental triggers, such as bacterial lipopolysaccharide (LPS), by the immune system play a critical role in both acute and chronic conditions. Monocyte and macrophage activation is demonstrably important in a number of diseases such as dry eye disease (DED) and periodontitis [1,2,3,4,5,6,7,8,9]. Innate immune signaling and activation of Toll-like receptors (TLRs) are increasingly thought to play a role in these chronic diseases. Anti-microbial peptides (AMPs) are multi-functional endogenous components of mucosal and other tissue defense systems that play a role in both innate immune signaling and disease processes. Synthesis and release of nitric oxide (NO) and other inflammatory cytokines such as IL-1β, IL-6, and TNF-α underpin the propagation of the immune response to environmental triggers. Cell-level regulation of these processes is driven by a number of signal transduction pathways, including NF-kB and MAPKs [1,10,11,12,13,14].

Histatins are an important family of endogenous anti-microbial peptides (AMPs) that are present in multiple human tissues and fluids, including the oral mucosa and ocular surface [15,16]. Histatin peptides have anti-bacterial and anti-fungal properties. Additional properties of histatins include pro-migratory actions, promotion of wound healing, immunomodulation, and dental enamel fortification [15,17,18]. Each member of this family of 13 peptides has relatively specialized functions and the physiologic effects of histatins may relate to the relative abundance of various members of the family in different pathologic states and tissues [15,19,20]. A number of investigators have sought to apply different functions of specific histatins to specific diagnostic and treatment applications. These applications range from anti-fungal studies, skin and ocular wound healing, ocular surface diseases, periodontitis, and the diagnosis of cancers [1,15,17,21,22,23]. Histatin-1 (Hst1) (sequence: DpSHEKRHHGYRRKFHEKHHSHREFPFYGDYGSNYLYDN) is an important member of the histatin family, and was one of the first histatins shown to be a wound-healing agent. Hst1 can promote epithelial wound healing in ocular, oral and skin applications. Hst1 and other histatins have been shown to neutralize LPS and reduce cytokine production related to the response to LPS in mucosal tissues, but their effects on immune cells are unknown [21,24]. Moreover, tear fluid levels of Hst1 have been inversely correlated with the diagnosis of severe DED [23].

Given the importance of macrophage activation in multiple disease processes related to the response to LPS, and the increasing awareness of the physiologic role of AMPs in multiple disease states, we sought to determine whether Hst1 could affect macrophage activation in RAW264.7 (RAW) cells.

## 2. Results

### 2.1. Histatin-1 Reduces LPS-Induced NO Production

**Figure 1:**
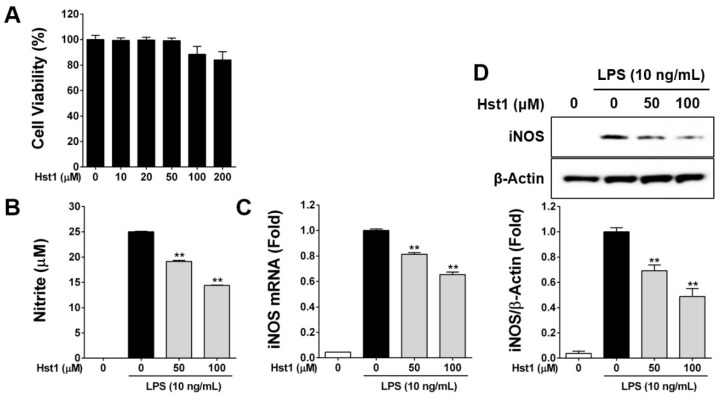
Effects of Hst1 on cell viability, nitric oxide production and iNOS expression in murine macrophage RAW264.7 cells. (**A**) Cell viability in HST1-treated cells was evaluated using the WST-1 assay. RAW264.7 cells were incubated with 10 to 200 μM of Hst1 for 18 h. The results are displayed as a percentage of control samples. The lack of Hst1 induced cell toxicity up to 200 μM is notable. Each value represents the mean ± SD and is representative of results obtained from three independent experiments. (**B**) Nitrite level was measured by the Griess assay. RAW264.7 cells were pre-treated with Hst1 at the indicated concentrations (50 and 100 μM) for 2 h and then exposed to Hst1 with LPS (10 ng/mL) or LPS alone for 16 h. The culture supernatants were subsequently isolated and analyzed for nitrite levels. Results reveal a statistically significant and dose-dependent reduction in LPS-induced nitrite production in Hst1-treated cells. The values for nitrite are the mean ± SD from three independent experiments. ** *p* < 0.01 compared to treatment with LPS alone. (**C**) For the qRT-PCR of iNOS gene expression, RAW264.7 cells were treated by the same procedure for Griess assay. The levels of iNOS mRNAs were determined by qRT-PCR analysis and GAPDH mRNA served as the internal control for the normalization of iNOS mRNA expression. The dose-dependent and statistically significant reduction in iNOS gene expression in cells treated with Hst1 is notable. (**D**) For the Western blotting of iNOS protein, lysates were prepared from RAW264.7 cells and treated by the same procedure for Griess assay. Total cellular proteins (40 μg) were resolved by SDS-PAGE, transferred to nitrocellulose membranes and detected with specific antibodies against murine iNOS and β-Actin served as the internal control for the normalization of iNOS protein expression. These results are displayed in fold of LPS alone samples. Results indicate that Hst1 treatment reduces protein levels of iNOS induced by LPS in a dose-dependent and statistically significant manner. Each value represents the mean ± SD and is representative of results obtained from three independent experiments. ** *p* < 0.01 compared to treatment with LPS alone.

To test whether Hst1 could reduce NO production by RAW cells, in response to LPS stimulation, we performed a series of experiments. First, we tested whether Hst1 was toxic to RAW cells and determined that Hst1 application did not demonstrate statistically significant toxicity up to 200 µM (Figure 1A). Next, we utilized a Griess assay to determine nitrite levels in RAW cells with and without Hst1 and with and without LPS treatment. This experiment revealed a dose-dependent reduction in nitrite produced in response to LPS by Hst1 treatment (Figure 1B) and was confirmed by testing the gene expression of iNOS using qRT-PCR. Figure 1C shows a dose-dependent reduction in LPS-induced iNOS expression in cells treated with increasing doses of Hst1. Finally, protein-level confirmation was performed with Western immunoblotting for iNOS protein. In Figure 1D, it is notable that the protein levels of iNOS, which were increased by LPS exposure of RAW cells, are reduced, in a dose-dependent manner, by treatment with Hst1.

### 2.2. Histatin-1 Reduces LPS-Induced Inflammatory Cytokine Production

**Figure 2:**
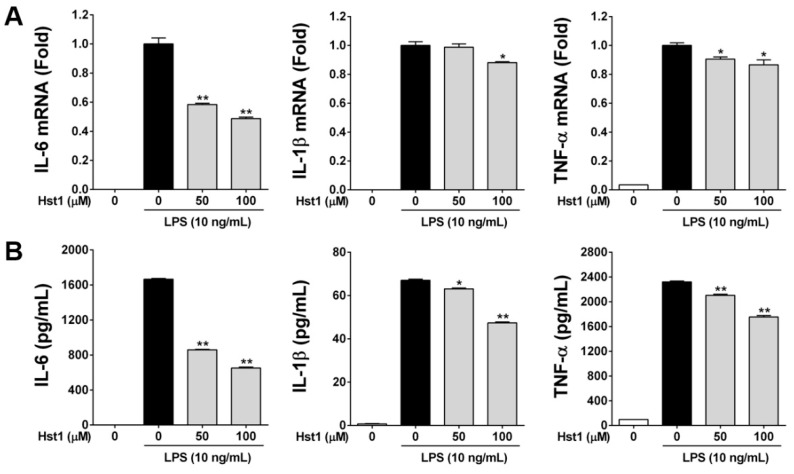
Effects of Hst1 on production of pro-inflammatory cytokines in LPS-stimulated RAW264.7 cells. (**A**) The mRNA levels of IL-6, IL-1β, and TNF-α were determined by using qRT-PCR. RAW264.7 cells were pre-treated with Hst1 at the indicated concentrations (50 and 100 μM) for 2 h and then exposed to Hst1 with LPS (10 ng/mL) or LPS alone for 16 h. The expression level of GAPDH mRNA served as the internal control for the normalization of IL-6, IL-1β, and TNF-α mRNAs expression. These results are displayed in fold of LPS alone samples. Gene expression analysis revealed a statistically significant reduction in inflammatory cytokine gene expression by treatment with Hst1. Each value represents the mean ± SD and is representative of results obtained from three independent experiments. * *p* < 0.05 and ** *p* < 0.01 compared to treatment with LPS alone. (**B**) RAW264.7 cells were treated by the same procedure for qRT-PCR. The production levels of IL-6, IL-1β, and TNF-α in the culture medium were determined by ELISA in triplicate samples according to the manufacturer’s instruction. The statistically significant, dose-dependent reduction of all inflammatory cytokine protein production by treatment with Hst1 is notable. Data are shown as the mean values ± SD (*n* = 3). * *p* < 0.05 and ** *p* < 0.01 compared to treatment with LPS alone.

In our next set of experiments, we sought to build upon the findings of reduced NO production in LPS-stimulated macrophages by determining whether similar effects were seen in LPS-induced inflammatory cytokine production. We first tested gene expression for IL-6, IL-1β, and TNF-α in RAW cells that were stimulated by LPS. All cytokines were significantly induced by LPS and showed a statistically significant reduction when treated with Hst1, with more pronounced effects seen in IL-6 as compared with IL-1β and TNF-α (Figure 2A). This gene-level assay was confirmed with protein-level assessment using ELISA. These assays revealed a statistically significant, dose-dependent, reduction in LPS-induced cytokine production by cells treated with Hst1 for IL-6, IL-1β, and TNF-α (Figure 2B).

### 2.3. Histatin-1 Reduces LPS-Induced MAPK Activation

**Figure 3:**
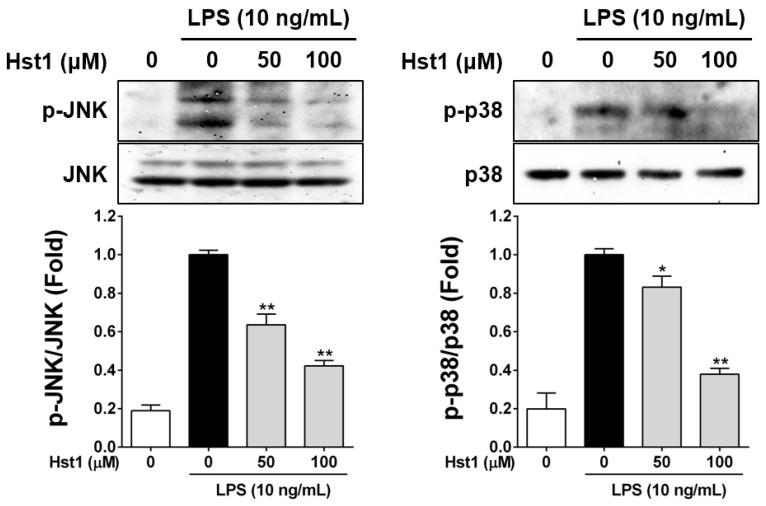
Effects of Hst1 on LPS-induced phosphorylation of MAPKs in RAW264.7 cells RAW264.7 cells were starved for 6 h and then pre-treated with HST1 at the indicated concentrations (50 and 100 μM) for 2 h and then exposed to HST1 with LPS (10 ng/mL) or LPS alone for 2 h. Whole-cell lysates were analyzed by Western blot analysis using specific phospho-JNK and phospho-p38 antibodies. The blot was stripped and reprobed with an antibody against non-phospho antibodies. Non phospho-JNK and p38 served as the control for the normalization of each MAPKs protein expression. These results are displayed in fold of LPS alone samples. The results of this analysis indicate that Hst1 treatment reduces LPS-induced phosphorylation of JNK and p38 in RAW cells by Hst1, in a dose-dependent manner. Each value represents the mean ± SD and is representative of results obtained from three independent experiments. * *p* < 0.05 and ** *p* < 0.01 compared to treatment with LPS alone.

After confirming that Hst1 could reduce LPS-stimulated NO and inflammatory cytokine production in RAW cells, we sought to test whether these effects could be underpinned by MAPK signaling modulation. We utilized Western immunoblotting for phosphorylated forms of two critical MAPK families related to inflammatory cellular signaling: JNK and p38. These experiments revealed that LPS treatment of RAW cells increased the p-JNK/total JNK and p-p38/total p38 ratios and that treatment with Hst1 reduced the abundance of the phosphorylated forms of these proteins in a dose-dependent, statistically significant manner (Figure 3).

### 2.4. Histatin-1 Reduces LPS-Induced NF-kB Activation

**Figure 4:**
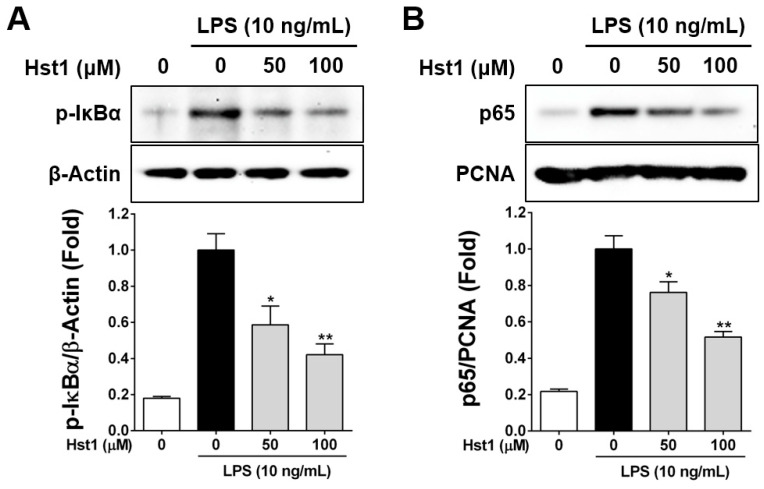
Effects of the Hst1 on the phosphorylation of IκB-α and nuclear translocation of NF-κB LPS-induced RAW264.7 macrophage cells. RAW264.7 cells were starved for 6 h, pre-treated with Hst1 at the indicated concentrations (50 and 100 μM) for 2 h and then exposed to Hst1 with LPS (10 ng/mL) or LPS alone for 2 h. (**A**) Whole-cell lysates were analyzed by Western blot analysis using a specific anti-phospho-IκB-α antibody. The blot was stripped and reprobed with an antibody against β-Actin. β-Actin was used as the internal control. Analysis reveals that p-IκB-α induction by LPS is reduced by treatment with Hst1 in RAW cells. (**B**) Nuclear extracts were prepared from RAW264.7 cells. RAW264.7 cells were treated by the same procedure for whole-cell lysates Western blot analysis and were analyzed using an anti-p65 antibody. The blot was stripped and reprobed with an antibody against PCNA to verify equal loading of proteins in each lane. These results are displayed in fold of LPS alone samples. Results indicate that the increased ratio of p65 to PCNA levels induced by LPS in RAW cells was statistically significantly reduced by treatment with Hst1. Each value represents the mean ± SD and is representative of results obtained from three independent experiments. * *p* < 0.05 and ** *p* < 0.01 compared to treatment with LPS alone.

Our final set of experiments were designed to determine whether NF-kB activation by LPS, in macrophages, was affected by treatment with Hst1. We tested this using Western blotting for the phosphorylated form of IκBα and determining the ratio of p65 to PCNA. In both of these assays, we determined that LPS-induced increases in p- IκBα and p65/PCNA levels were reduced by treatment with Hst1 in a statistically significant and dose-dependent manner (Figure 4).

## 3. Discussion

In this study, we sought to determine whether Hst1 could reduce environmental trigger-induced inflammatory signaling and cytokine production in macrophages. Our results indicate that LPS-induced inflammation markers in RAW cells were substantially reduced by treatment with Hst1. These results have important implications in our understanding of the effects of this important AMP in macrophage responses to bacterial components.

Responses to environmental triggers, such as LPS, and innate immune activation are increasingly seen to have critical roles in acute and multiple chronic disease processes, such as ocular surface diseases, DED, and periodontitis, amongst others. Macrophages are critical players in responding to these triggers and propagating inflammatory responses. NO and inflammatory cytokines, such as IL-6, IL-1β, and TNF-α, underpin many of the desirable and undesirable consequences of immune responses to environmental triggers [1].

AMPs, such as histatins, have been studied for their physiological roles in mucosal and non-mucosal disease states and are increasingly thought to be important homeostatic elements with multiple immunomodulatory and epithelial trophic activities. Hst1 in particular is thought to interact with the host system in multiple ways, including wound healing and pro-migratory properties [1,15,21,22]. Hst1 has also been shown to inhibit hemolysis of red blood cells by LPS [24]. Multiple AMPs are known to exhibit LPS neutralizing activity. In fact, a derivative of histatin-5 (Hst5), called P-113, has been tested as a potential anti-microbial therapeutic and for its LPS neutralizing activity [25]. Similarly, the AMP LL-37 has been demonstrated to reduce IL-6 and IL-8 expression in gingival tissues in response to bacterial components [26]. AMPs may have roles to play in the treatment of chronic diseases such as periodontitis and DED and some of these potential applications may be dependent on the effects of AMPs on immune cells such as macrophages. Modulation of polarization of macrophages and alterations in the homeostasis of macrophage activity is critically important to disease pathogenesis [2,7,8,9].

Understanding the roles of AMPs in modulating the immune response to bacterial components is useful in elucidating their role in both acute and chronic disease processes. Moreover, as has been increasingly attempted, a better understanding of the effects of AMPs in diverse tissue types can be leveraged for drug development. The results of this study provide initial evidence of the impact of histatin-1 on macrophages and build upon findings that histatin-1 can have immune modulatory properties, and that its loss has been associated with inflammatory conditions [23,27]. While the results of these experiments show the consistent anti-inflammatory properties of Hst1 in RAW cells exposed to LPS, future testing in human tissues and in vivo will be necessary to determine whether these findings can be applied more broadly or built upon for therapeutic application.

## 4. Materials and Methods

### 4.1. Materials and Cell Culture

Hst1 was purchased from the Northwestern University Peptide Synthesis Core Facility (Evanston, IL, USA). LPS was purchased from Sigma-Aldrich (Lipopolysaccharides from *Escherichia coli* O111:B4, St. Louis, MO, USA). Antibodies for β-Actin were obtained from Santa Cruz Biotechnology (Dallas, TX, USA). Specific antibodies against iNOS (NOS2), phospho-JNK, JNK, phospho-p38, p38, phospho-IκBα, p65 and proliferating cell nuclear antigen (PCNA) were obtained from Cell Signaling Technology (Danvers, MA, USA). The murine macrophage RAW264.7 cell line, purchased from American Type Culture Collection (ATCC, Manassas, VA, USA), was cultured in Dulbecco’s modified Eagle’s medium (DMEM, Gibco, Grand Island, NY, USA) containing 10% fetal bovine serum (FBS, Gibco). Cells were incubated with 10 ng/mL LPS along with various concentrations of Hst1 for 2 h to 18 h, as indicated. For all experiments, except for WST-1 viability assays, cells were pre-treated with Hst1 for 2 h and then treated with Hst1 with LPS, with details noted below.

### 4.2. Viability of RAW264.7 Macrophage Cells

RAW264.7 cells (purchased directly from ATCC solely for the included experiments) were incubated with 10 to 200 μM of Hst1 in a serum-free medium for 18 h. Cells were then treated with 10 μL of WST-1 (Roche, Mannheim, Germany) for 30 min. The percentage of cell viability was calculated as follows: (mean optical density (OD) in HST1-treated cells/mean OD in untreated cells × 100).

### 4.3. Measurement of NO Production

RAW264.7 cells were treated with LPS in the presence or absence of Hst1 (2 h pretreatment with Hst1 and then co-treatment with LPS) for the indicated times. NO production was analyzed using the Griess Reagent System (Sigma).

### 4.4. Quantitative Real-Time Polymerase Chain Reaction (PCR)

RAW264.7 cells were treated with LPS with or without Hst1 (2 h pretreatment with Hst1 and then co-treatment with LPS) for the indicated times. To evaluate the expression levels of iNOS, IL-6, IL-1β, and TNF-α mRNA, total RNA was prepared with an RNeasy Plus Mini Kit (Qiagen, Hilden, Germany) according to the manufacturer’s instructions. The following primers were used: iNOS (Mm00440485_m1), IL6 (Mm00446190_m1), IL-1β (Mm00434228_m1), TNF-α (Mm00443258_m1), and GAPDH (Mm99999915_g1). GAPDH mRNA levels were used as internal controls. A negative control without a cDNA template was performed. The fold increase was determined relative to a control after normalizing to GAPDH using the 2^−ΔΔCT^ method.

### 4.5. Western Blot Analysis

RAW264.7 cells were cultured and stimulated with LPS with or without Hst1 (2 h pretreatment with Hst1 and then co-treatment with LPS) for the indicated times. T Protein concentration was quantified (Bio-Rad Laboratories, Hercules, CA, USA). The cells were prepared using a Nuclear Extraction Kit (Abcam, Cambridge, UK). Proteins were separated on NuPAGE 10% Bis-Tris gel (Invitrogen, Carlsbad, CA, USA)). Nonspecific binding was blocked by soaking the membrane in a Tris-buffered saline containing 3–5% non-fat dry milk (Santa Cruz). The membranes were reacted with primary antibodies against iNOS, p-JNK, JNK, p-p38, p38, p-IκBα, p65, PCNA, and β-Actin. Horseradish peroxide-conjugated secondary antibodies were detected using enhanced chemiluminescence detection (Amersham Bioscience, Buckinghamshire, UK). Protein expression was determined by the analysis of the signals captured using an image analyzer (myECL, Thermo Scientific, Waltham, MA, USA).

### 4.6. Enzyme-Linked Immunosorbent Assay (ELISA)

Cytokines IL-6, IL-1β, and TNF-α were measured using ELISA kits (R&D Systems, Minneapolis, MN, USA). RAW264.7 cells were cultured for 24 h, washed with DPBS (Gibco), and grown in a serum-free medium containing various concentrations of Hst1 (2 h pretreatment with Hst1 and then co-treatment with LPS). Two hours after pretreatment with the Hst1, RAW274.7 cells were stimulated with 10 ng/mL of LPS for 16 h and supernatants were collected for cytokine measurement.

### 4.7. Statistical Analysis

Unless otherwise stated, all experiments were performed with triplicate samples and repeated at least three times. The results are expressed as the mean ± standard deviation (SD) and analyzed using one-way analysis of variance (ANOVA) followed by Dunnett’s tests for multiple comparisons or unpaired Student’s *t*-tests for two-group comparisons. All analyses were performed using Prism 6.0 (GraphPad Software, San Diego, CA, USA), and *p*-values < 0.05 were considered statistically significant.

## 5. Conclusions

Thus, Hst1 reduces inflammatory signaling, NO production and immune cell signaling related to LPS induction in RAW cells. These results have implications for our understanding of the effects of AMPs in immune cells and inflammatory signaling.

## Data Availability

The data that support the findings of this study are available from the corresponding author upon reasonable request, and upon clearance from the University of Illinois at Chicago and/or the Veterans Affairs Administration. The data presented herein do not represent the views of the National Institutes of Health, Veterans Affairs or Department of Defense.

## References

[1] Eshac Y., Redfern R.L., Aakalu V.K. (2021). The Role of Endogenous Antimicrobial Peptides in Modulating Innate Immunity of the Ocular Surface in Dry Eye Diseases. Int. J. Mol. Sci..

[2] Pussinen P.J., Vilkuna-Rautiainen T., Alfthan G., Palosuo T., Jauhiainen M., Sundvall J., Vesanen M., Mattila K., Asikainen S. (2004). Severe periodontitis enhances macrophage activation via increased serum lipopolysaccharide. Arterioscler. Thromb. Vasc. Biol..

[3] Simmons K.T., Xiao Y., Pflugfelder S.C., de Paiva C.S. (2016). Inflammatory response to lipopolysaccharide on the ocular surface in a murine dry eye model. Investig. Ophthalmol. Vis. Sci..

[4] You I.-C., Coursey T.G., Bian F., Barbosa F.L., de Paiva C.S., Pflugfelder S.C. (2015). Macrophage phenotype in the ocular surface of experimental murine dry eye disease. Arch. Immunol. Ther. Exp..

[5] Zhou D., Chen Y.-T., Chen F., Gallup M., Vijmasi T., Bahrami A.F., Noble L.B., van Rooijen N., McNamara N.A. (2012). Critical Involvement of Macrophage Infiltration in the Development of Sjögren’s Syndrome–Associated Dry Eye. Am. J. Pathol..

[6] Stevenson W., Chauhan S.K., Dana R. (2012). Dry eye disease: An immune-mediated ocular surface disorder. Arch. Ophthalmol..

[7] Garaicoa-Pazmino C., Fretwurst T., Squarize C.H., Berglundh T., Giannobile W.V., Larsson L., Castilho R.M. (2019). Characterization of macrophage polarization in periodontal disease. J. Clin. Periodontol..

[8] Yang J., Zhu Y., Duan D., Wang P., Xin Y., Bai L., Liu Y., Xu Y. (2018). Enhanced activity of macrophage M1/M2 phenotypes in periodontitis. Arch. Oral Biol..

[9] Almubarak A., Tanagala K.K.K., Papapanou P.N., Lalla E., Momen-Heravi F. (2020). Disruption of monocyte and macrophage homeostasis in periodontitis. Front. Immunol..

[10] Redfern R.L., Barabino S., Baxter J., Lema C., McDermott A.M. (2015). Dry eye modulates the expression of toll-like receptors on the ocular surface. Exp. Eye Res..

[11] Redfern R.L., McDermott A.M. (2010). Toll-like receptors in ocular surface disease. Exp. Eye Res..

[12] Redfern R.L., Patel N., Hanlon S., Farley W., Gondo M., Pflugfelder S.C., McDermott A.M. (2013). Toll-like receptor expression and activation in mice with experimental dry eye. Investig. Ophthalmol. Vis. Sci..

[13] Redfern R.L., Reins R.Y., McDermott A.M. (2011). Toll-like receptor activation modulates antimicrobial peptide expression by ocular surface cells. Exp. Eye Res..

[14] Reins R.Y., Courson J., Lema C., Redfern R.L. (2017). MyD88 contribution to ocular surface homeostasis. PLoS ONE.

[15] Torres P., Castro M., Reyes M., Torres V. (2018). Histatins, wound healing, and cell migration. Oral Dis..

[16] Shah D., Ali M., Pasha Z., Jaboori A.J., Jassim S.H., Jain S., Aakalu V.K. (2016). Histatin-1 expression in human lacrimal epithelium. PLoS ONE.

[17] Shah D., Ali M., Shukla D., Jain S., Aakalu V.K. (2017). Effects of histatin-1 peptide on human corneal epithelial cells. PLoS ONE.

[18] Shah D., Son K.-N., Kalmodia S., Lee B.-S., Ali M., Balasubramaniam A., Shukla D., Aakalu V.K. (2020). Wound Healing Properties of Histatin-5 and Identification of a Functional Domain Required for Histatin-5-Induced Cell Migration. Mol. Ther. Methods Clin. Dev..

[19] Van Dijk I.A., Veerman E.C., Reits E.A., Bolscher J.G., Stap J. (2018). Salivary peptide histatin 1 mediated cell adhesion: A possible role in mesenchymal-epithelial transition and in pathologies. Biol. Chem..

[20] Torres P., Díaz J., Arce M., Silva P., Mendoza P., Lois P., Molina-Berríos A., Owen G.I., Palma V., Torres V.A. (2017). The salivary peptide histatin-1 promotes endothelial cell adhesion, migration, and angiogenesis. FASEB J..

[21] Gusman H., Travis J., Helmerhorst E.J., Potempa J., Troxler R.F., Oppenheim F.G. (2001). Salivary histatin 5 is an inhibitor of both host and bacterial enzymes implicated in periodontal disease. Infect. Immun..

[22] Khurshid Z., Najeeb S., Mali M., Moin S.F., Raza S.Q., Zohaib S., Sefat F., Zafar M.S. (2017). Histatin peptides: Pharmacological functions and their applications in dentistry. Saudi Pharm. J..

[23] Kalmodia S., Son K.-N., Cao D., Lee B.-S., Surenkhuu B., Shah D., Ali M., Balasubramaniam A., Jain S., Aakalu V.K. (2019). Presence of histatin-1 in human tears and association with aqueous deficient dry eye diagnosis: A preliminary study. Sci. Rep..

[24] Sugiyama K. (1993). Anti-lipopolysaccharide activity of histatins, peptides from human saliva. Experientia.

[25] Chih Y.-H., Wang S.-Y., Yip B.-S., Cheng K.-T., Hsu S.-Y., Wu C.-L., Yu H.-Y., Cheng J.-W. (2019). Dependence on size and shape of non-nature amino aciD.S. in the enhancement of lipopolysaccharide (LPS) neutralizing activities of antimicrobial peptides. J. Colloid Interface Sci..

[26] Inomata M., Into T., Murakami Y. (2010). Suppressive effect of the antimicrobial peptide LL-37 on expression of IL-6, IL-8 and CXCL10 induced by Porphyromonas gingivalis cells and extracts in human gingival fibroblasts. Eur. J. Oral Sci..

[27] Zheng Y., Yuan W., Liu H., Huang S., Bian L., Guo R. (2020). Injectable supramolecular gelatin hydrogel loading of resveratrol and histatin-1 for burn wound therapy. Biomater. Sci..

